# Accident and Injury

**Published:** 1899-02

**Authors:** 


### Book Reviews.

ACCIDENT AND INJURY. Their Relations to Diseases of the Nervous System. By PEARCE BAILEY, A. M., M. D. New York: D. Appleton & Company. 1898

The subject of traumatic nervous affections has received considerable attention by the School of Salpêtriére, in France, by Oppenheim and others in Germany, and by Knapp, Clevenger and others in his country. To these must now be added the splendid production of Pearce Bailey, which traverses the whole subject in a thoroughly scientific, able and liberal manner. The importance of this subject is extremely great, when one recalls the fact that case after case of traumatic nervous disease finds its way into the courts where the utmost care must be exercised in the correct interpretation and presentation of the symptoms and facts by the attending physician. The author divides the work into four parts as follows :

The introduction treats of the accident, the previous history of the patient, the physical evidences of predisposition to nervous disease, and the examination for the actual injury.

Part I. treats of the organic effects of injury to the nervous system, such as injuries to the brain, spinal cord and peripheral nerves. The ultimate organic effects of injury, as epilepsy, general paralysis of the insane, locomotor ataxia, progressive muscular atrophy and paralysis agitans are then taken up separately.

Part II. treats of the functional effects of injury, known generally as the traumatic neuroses, which includes traumatic hysteria, traumatic neurasthenia, and some unclassified forms.

In Part III. the author takes up the difficult question of malingering and treats it with much tact and clearness. The exaggeration of the symptoms actually present is a very difficult matter to decide, also the simulation practised by adept individuals. Part IV. takes up the question of treatment, considering the use of electricity, massage, rest cure, hydrotherapy, isolation and hypnotism. A carefully prepared index of names and subject-matter completes the volume. Fifty-five well executed illustrations accompany the text. On the whole, no better work in the English language has appeared on this subject and the author is to be congratulated on his success. The book-making, paper, print and binding are unexcelled.

